# MALAT1 Expression Is Deregulated in miR-34a Knockout Cell Lines

**DOI:** 10.3390/ncrna11040060

**Published:** 2025-08-05

**Authors:** Andrea Corsi, Tonia De Simone, Angela Valentino, Elisa Orlandi, Chiara Stefani, Cristina Patuzzo, Stefania Fochi, Maria Giusy Bruno, Elisabetta Trabetti, John Charles Rotondo, Chiara Mazziotta, Maria Teresa Valenti, Alessandra Ruggiero, Donato Zipeto, Cristina Bombieri, Maria Grazia Romanelli

**Affiliations:** 1 Department of Neurosciences, Biomedicine and Movement Sciences, University of Verona, Strada le Grazie 8, 37134 Verona, Italy; andrea.corsi@univr.it (A.C.); tonia.desimone@univr.it (T.D.S.); angela.valentino@univr.it (A.V.); elisa.orlandi@univr.it (E.O.); chiara.stefani@univr.it (C.S.); cristina.patuzzo@univr.it (C.P.); stefania.fochi@univr.it (S.F.); mariagiusy.bruno@univr.it (M.G.B.); elisabetta.trabetti@univr.it (E.T.); mariateresa.valenti@univr.it (M.T.V.); alessandra.ruggiero@univr.it (A.R.); donato.zipeto@univr.it (D.Z.); 2 IRCSS Ospedale Policlinico San Martino, Largo Rosanna Benzi 10, 16132 Genova, Italy; johncharles.rotondo@hsanmartino.it; 3 Department of Medical Sciences, University of Ferrara, 44121 Ferrara, Italy; mzzchr@unife.it

**Keywords:** miR-34a, hsa-miR-34a, CRISPR, gene editing, miRNA, lncRNA, MALAT1

## Abstract

**Background/Objectives:** Non-coding microRNA-34a (miR-34a) regulates the expression of key factors involved in several cellular processes, such as differentiation, apoptosis, proliferation, cell cycle, and senescence. Deregulation of the expression of these factors is implicated in the onset and progression of several human diseases, including cancer, neurodegenerative disorders, and pathologies associated with viral infections and inflammation. Despite numerous studies, the molecular mechanisms regulated by miR-34a remain to be fully understood. The present study aimed to generate miR-34a knockout cell lines to identify novel genes potentially regulated by its expression. **Methods:** We employed the CRISPR-Cas9 gene editing system to knock out the hsa-miR-34a gene in HeLa and 293T cell lines, two widely used models for studying molecular and cellular mechanisms. We compared proliferation rates and gene expression profiles via RNA-seq and qPCR analyses between the wild-type and miR-34a KO cell lines. **Results:** Knockout of miR-34a resulted in a decreased proliferation rate in both cell lines. Noteworthy, the ablation of miR-34a resulted in increased expression of the long non-coding RNA MALAT1. Additionally, miR-34a-5p silencing in the A375 melanoma cell line led to MALAT1 overexpression. **Conclusions:** Our findings support the role of the miR-34a/MALAT1 axis in regulating proliferation processes.

## 1. Introduction

Micro-RNAs (miRNAs) are small non-coding RNAs that modulate gene expression preferentially by binding the 3′-UTR region of the target messenger RNA. MiRNA-mediated control of gene expression is critical for cellular response to environmental stress, such as starvation, hypoxia, oxidative stress, and DNA damage. A considerable amount of evidence correlates altered miRNA expression to human diseases affecting cell proliferation, survival, and tissue differentiation [1]. A set of miRNAs are functionally classified as oncogenes, referred to as “oncomiRs” or tumor suppressor miRs, whose dysregulation is associated with cancer initiation, progression, and metastasis [2,3]. The miR-34 family, along with the let-7 and miR-200 families, represent the three major families of tumor-suppressive miRNAs [4]. The miR-34 family consists of three members, miR-34a, miR-34b, and miR-34c, which are expressed by two different genes. miR-34a is transcribed from a gene located on chromosome 1p36.22, while a polycistronic transcript from chromosome 11q23.1 expresses miR-34b and c [4]. miR-34a, miR-34b, and miR-34c share a high degree of homology, which may account for overlapping functions in gene expression regulation [5,6]. miR-34a is more highly expressed in human tissues than miR-34b/c, excluding lungs, where miR-34b and c predominate [6]. The miR-34 family has been demonstrated to play a pivotal role in the regulation of apoptosis, cell cycle, and senescence, targeting multiple oncogenic mRNAs, such as CDK4, CDK6, BCL2 apoptosis regulator (BCL2), sirtuin 1 (SIRT1), and cyclin D1 (CCND1) genes [7,8,9]. CpG methylation has been demonstrated to silence the expression of miR-34 family members in various cancers [10]. miR-34a expression is regulated by the tumor suppressor p53 and is frequently downregulated in several cancers [11]. miR-34a may act as a tumor suppressor by negatively regulating the cell cycle [10], epithelial–mesenchymal transition (EMT) suppression [12], and p53-mediated induction of apoptosis [13,14]. In lung cancer, miR-34a has been demonstrated to contribute to increased cell proliferation, metastasis, and invasion [10,15,16].

In this study, we aimed to generate and characterize two independent miR-34a knockout (miR-34a KO) cell lines via CRISPR/Cas9 genetic engineering to identify novel miR-34a targets. Taking advantage of high-throughput transcriptomic analysis, we found that the expression of MALAT1, a long non-coding RNA (lncRNA), is altered in both miR-34a KO cell lines, suggesting a possible interplay in the miR-34a/MALAT1 axis in promoting cellular proliferation.

## 2. Results

### 2.1. Generation and Characterization of miR-34a KO HeLa and 293T Cell Lines

**Figure 1:**
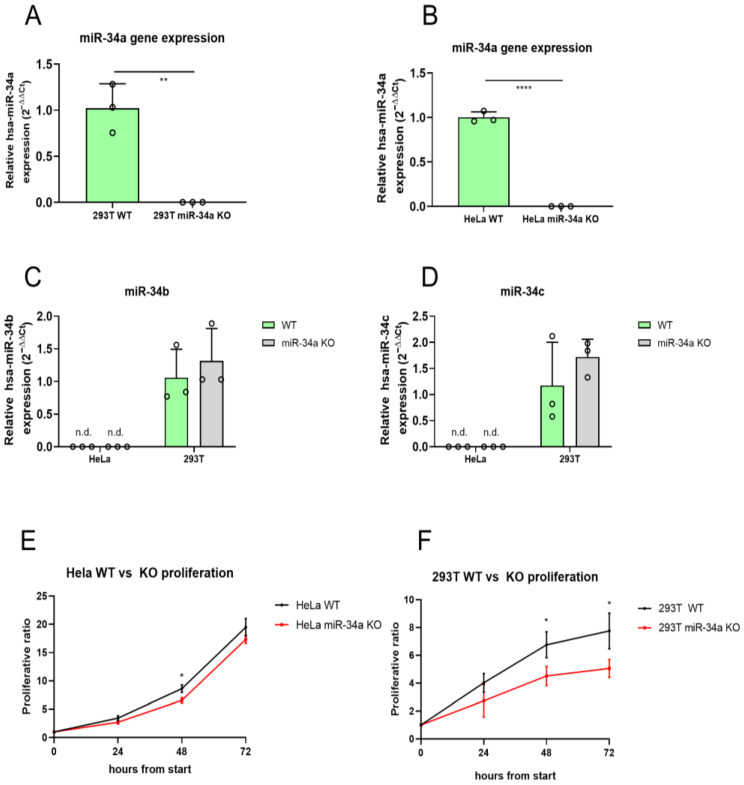
miR-34a expression and cell proliferation evaluation in HeLa and 293T WT and KO cells. qPCR analysis of miR-34a-5p gene expressions in HeLa (**A**) and 293T (**B**) WT and KO cell lines. qPCR analysis of miR-34b (**C**) and miR-34c (**D**) expression in HeLa and 293T WT and KO cell lines. Proliferation curves of WT versus KO in HeLa (**E**) and 293T (**F**) cells measured as a fold change relative to timepoint 0 using CCK-8 assay. * = *p*-value ≤ 0.05; ** = *p*-value ≤ 0.01; **** = *p*-value ≤ 0.0001; n.d. = not detectable; WT = wild-type for miR-34a; KO = knockout for miR-34a; *n* = 3 for each experimental group, except 293T miR-34a KO cells in the proliferation analysis (*n* = 4).

A CRISPR/Cas9-mediated gene editing approach was used to generate HeLa and 293T miR-34a-KO cell lines. We selected three gRNAs taking advantage of the bioinformatics tool CHOPCHOP v3 [17]. All three selected guide RNAs recognized regions in the pre-miR-34a (Figure S1). We checked potential off-targets of the three gRNAs and verified that none reside in the coding gene sequences (Table S1). The knockout of the miR-34a gene was validated by Sanger sequencing, end-point PCR (Figure S2), and RT-qPCR using TaqMan probes specific for miR-34a-5p (Figure 1A,B). To ascertain which miR-34 family members were expressed in both cell lines, we also analyzed the expression levels of miR-34b and miR-34c in the wild-type (WT) and KO cell lines. RT-qPCR analyses demonstrated no significant differences in the expression of both miR-34b and miR-34c between 293T WT and KO cell lines, while HeLa cells did not display detectable levels of either transcript (Figure 1C,D). These results suggest that the HeLa miR34-a KO cell model may be applied as an in vitro cell model to investigate the essential role of all members of the miR-34 family.

### 2.2. Loss of miR-34a Alters Cellular Proliferation in 293T Cells

CCK-8 proliferation assay was used to measure the proliferative rate of both WT and KO cell lines over a period of 72 h. miR-34a KO HeLa cells showed a reduced proliferation rate effect compared to WT cells, detectable at 48 h. Depletion of miR-34ain 293T cells led to a reduction in the proliferation rate at 48 and 72 h compared to WT cells. These results suggest that the absence of miR-34a affects cell cycle progression and survival (Figure 1E,F).

### 2.3. RNA-Seq Analysis Identifies Genes Deregulated in HeLa miR-34a KO Cells Compared to WT

Based on the qPCR results showing no detectable expression of miR-34b and miR-34c in the HeLa cell line, we select to perform RNA-seq analysis on miR-34a KO HeLa cells and compare transcripts expression to WT HeLa cells. miR-34a KO HeLa may serve as a suitable model to highlight the essential role of miR-34 family members due to the lack of all three different miR-34s. Transcriptomic analysis revealed 185 genes differentially expressed (logFC > |0.5| and *p*-value < 0.05), of which 82 were downregulated and 103 were upregulated (Supplementary Figure S3A–C).

Gene Ontology (GO) analysis of the differentially expressed genes identified an enrichment in GO terms regarding biological processes generally associated with neural cells, including axon guidance, synapse assembly, and neuron projection guidance (Supplementary Figure S4A–C). KEGG (Kyoto Encyclopedia of Genes and Genomes) analysis identified pathways related to cancer, specifically small cell lung cancer, and cardiomyopathies, including arrhythmogenic right ventricular cardiomyopathy, cytoskeleton in muscle cells and hypertrophic cardiomyopathy (Supplementary Figure S4D).

We chose to validate the overexpression by qPCR of five of the most significantly upregulated genes identified by RNA-seq: LINC03057, MALAT1 (Metastasis Associated Lung Adenocarcinoma Transcript 1), MAP1B (Microtubule-Associated Protein 1B), ONECUT2 and REL, based on their relevance in proliferative processes and cancer.

LINC03057, also known as lnc-HOXB8-1:2 (ENSG00000272763), is an oncogenic lncRNA associated with colorectal cancer progression [18]. MALAT1, also known as LINC00047 (ENSG00000251562) is a lncRNA involved in triple-negative breast cancer [19], B-cell lymphoma [20], colorectal cancer [21] and multiple myeloma [22]. MAP1B (ENSG00000131711), is a microtubule-associated protein involved in several types of cancer, including non-small cell lung cancer [23], urothelial carcinoma [24], and triple-negative breast cancer [25]. ONECUT2, also known as One Cut Homeobox 2 (ENSG00000119547), is a transcription factor that upregulates cell proliferation, migration, adhesion, and differentiation processes in several tumors, including prostate, colorectal, ovarian, and lung cancer [26]. REL, also known as c-REL (ENSG00000162924), is a subunit of the Nuclear Factor kB (NF-kB) complex involved in inflammation processes, in liquid tumors (including Hodgkin’s lymphoma, Adult T-cell leukemia/lymphoma, marginal zone lymphoma) and solid tumors (including breast cancer, pancreatic cancer, gastric cancer) [27].

**Figure 2:**
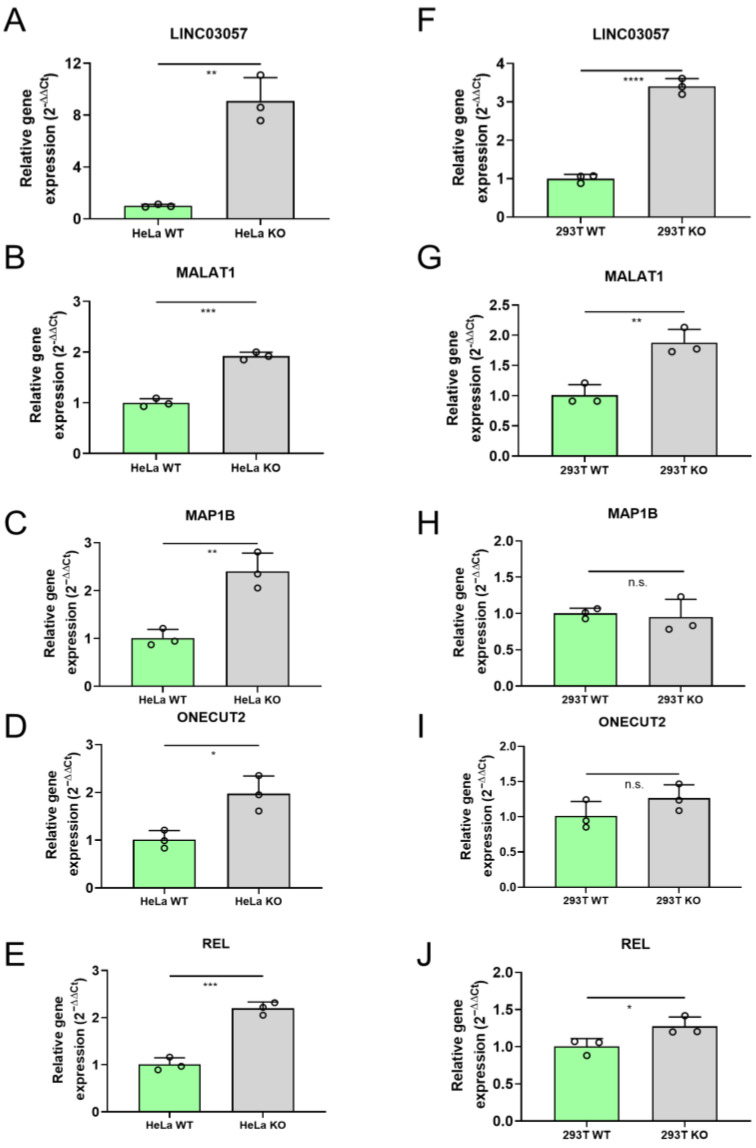
RT-qPCR analysis of LINC03057, MALAT1, MAP1B, ONECUT2, and REL mRNA expression in miR-34a KO compared to WT cells. mRNAs expression levels in HeLa WT vs. miR-34a KO (**A**–**E**); mRNAs expression in 293T WT vs. miR-34a KO (**F**–**J**). * = *p*-value ≤ 0.05; ** = *p*-value ≤ 0.01; *** = *p*-value ≤ 0.001; **** = *p*-value ≤ 0.0001; n.s. = not statistically significant; *n* = 3 for each experimental group.

Quantitative PCR analysis confirmed the upregulation of LINC03057, MALAT1, MAP1B, ONECUT2, and REL in HeLa KO cells (Figure 2A–E).

Next, we assessed whether these genes were also upregulated in the 293T KO cell line. We found that REL, MALAT1, and LINC03057 were significantly upregulated, while MAP1B and ONECUT2 did not display significant differences in expression compared to WT (Figure 2F–J). These results suggest that LINC03057, MALAT1, and REL could be potential targets of post-transcriptional expression regulation by miR-34a.

### 2.4. Rescue of miR-34a Expression in 293T miR34-a KO Inhibits MALAT1 Expression

**Figure 3:**
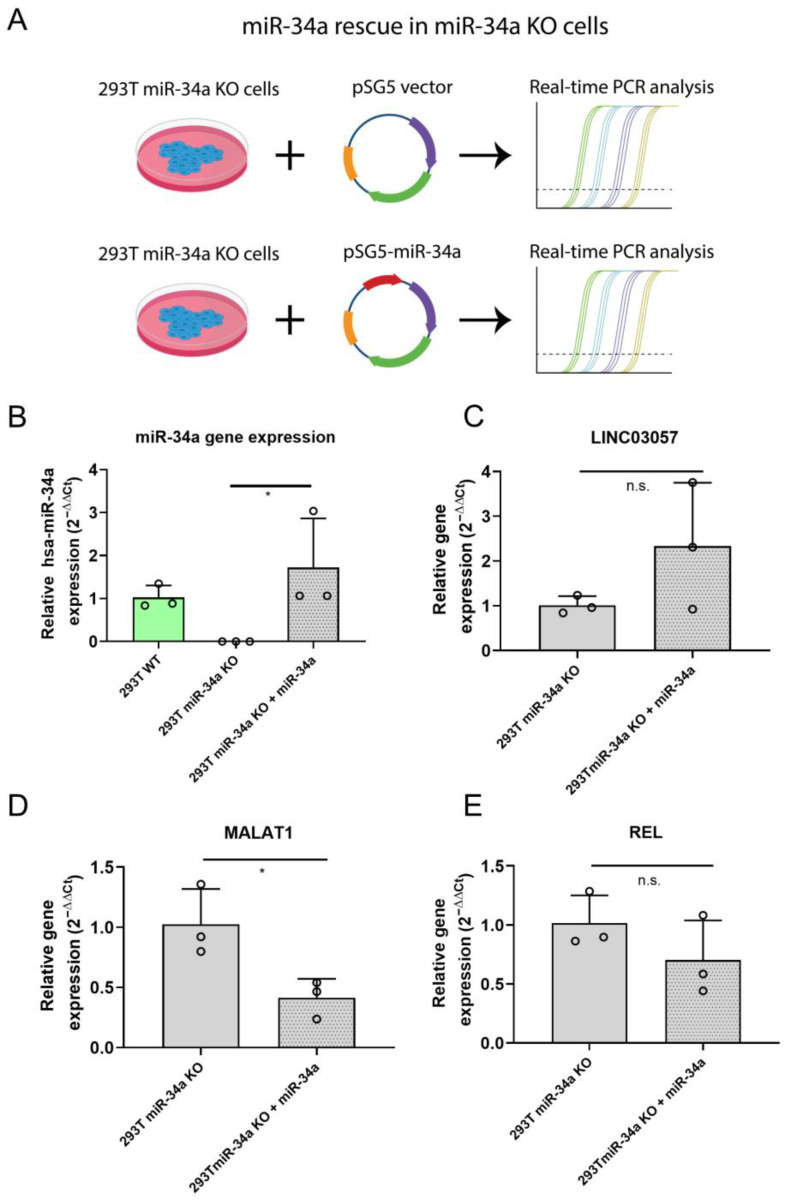
Analysis of LINC03057, MALAT1 and REL mRNA expression in 293T miR-34a KO cells rescued for miR-34a expression. (**A**) Graphical scheme summarizing the experimental design for miR-34a rescue in 293T miR-34a KO cells. (**B**) qPCR analysis of miR-34a-5p gene expression in 293T WT, 293T miR-34a KO and 293T miR-34a KO cells transfected with an miR-34a overexpressing vector; (**C**–**E**) RT-qPCR analysis of LINC03057 (**C**), MALAT1 (**D**) and REL (**E**) mRNA expression in miR-34a KO cells compared to miR-34a KO cells rescued for miR-34a expression. * = *p*-value ≤ 0.05; n.s. = not statistically significant; *n* = 3 for each experimental group.

To further investigate the effect of miR-34a expression on LINC03057, MALAT1, and REL transcripts, 293T miR34-a KO cells were transfected with a vector expressing miR-34a (pSG5-miR34a) or the corresponding empty vector as control (pSG5) (Figure 3A).

RT-qPCR analysis showed that miR-34a overexpression led to a statistically significant downregulation of MALAT-1 (Figure 3D), whereas the effect on LINC03057 and REL (Figure 3C,E) was not statistically significant.

These results corroborate the hypothesis that miR-34a may participate in the regulation of MALAT1 gene expression.

### 2.5. miR-34a Inhibition in A375 Melanoma Cells Affects MALAT1 Expression

It has been previously demonstrated that MALAT1 is able to reduce miR-34a action in melanoma cells by acting as a “sponge” via its direct binding to miR-34a [28]. However, the possibility of miR-34ato counter-regulating MALAT1 expression is currently unexplored. Therefore, we assessed whether the miR-34a expression affected MALAT1 expression by inhibiting miR-34a-5p in A375 melanoma cells.

**Figure 4:**
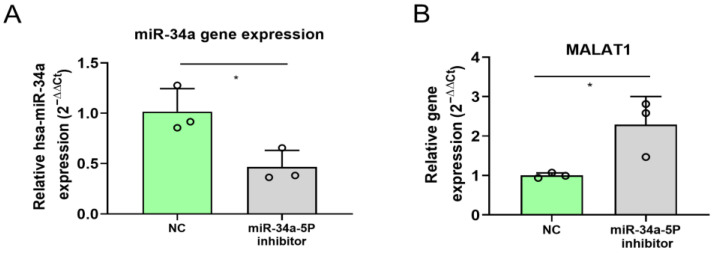
Analysis of MALAT1 expression levels in melanoma A375 cells in presence of miR-34a-5p inhibition. (**A**) qPCR analysis of relative miR-34a-5p gene expression in A375 melanoma cells transfected with scramble (NC) or miR-34a-5p inhibitor, showing reduced expression of miR-34a-5p after inhibition. (**B**) RT-qPCR analysis of MALAT1 in A375 melanoma cells transfected with scramble (NC) or miR-34a-5p inhibitor. * = *p*-value ≤ 0.05; *n* = 3 for each experimental group.

The result showed that the inhibition of miR-34a-5p induced overexpression of MALAT1 (Figure 4A,B), suggesting that, in the miR-34a/MALAT1 regulatory axis, the microRNA may alter the gene expression of the lncRNA MALAT1.

## 3. Discussion

The miRNA-34 family regulates several signaling pathways associated with the immune system, metabolism, cellular structure, and cell cycle progression by targeting specific mRNAs whose expression is modulated at both transcriptional and post-transcriptional levels [9]. Since hundreds of targets are envisaged to be regulated by the miR-34 family, their role in several diseases is not surprising. Members of the miR-34 family have been shown to participate in tumor suppression, such as colorectal, breast, prostate, lung, liver cancer, hematological neoplasm, and osteosarcoma [29], but also in neurological and depressive disorders, and in stress-related psychiatric conditions [30,31,32,33]. miR-34a is one of the miRNAs with the most significant expression regulation mediated by p53-, and displays tumor suppressor properties [10]. In fact, miR-34a can negatively affect cell cycle progression [10], block EMT [12], and induce p53-mediated apoptosis [13,14] through the regulation of its downstream target genes.

Due to the complexity of the molecular mechanisms that may be affected by miR-34a, specific KO cell lines provide a useful in vitro model to interpret the essential role of miR-34a and open the investigation to what processes may be circumvented by other miR-34 members. Here, we characterized two cell lines KO for miR-34a, analyzing proliferation rates and transcript expression.

Gene Ontology analysis of the differentially expressed genes in the miR-34a KO HeLa cells developed in the present study identified enrichment in biological processes associated with neural cells. Interestingly, miR-34a is highly expressed in the human brain, and has been associated with a wide range of neurodevelopmental and neuropathological processes [34]. Moreover, in recent years, Hu and colleagues demonstrated that miR-34a promotes dendritic growth and branching in cultured hippocampal neurons [35]. The KEGG v3 analysis identified pathways related not only to cancer but also to cardiomyopathies. These results are consistent with early reports demonstrating miR-34a role in cardiac functionality, heart and skeletal muscle aging, and cardiomyopathies [36,37,38,39,40], thus proposing miR-34a as a novel therapeutic target for treating cardiovascular diseases [41].

Transcript expression analysis performed on miR-34a KO HeLa cells revealed the deregulation of several genes. In particular, three of them (LINC03057, MALAT1, and REL) resulted upregulated in both HeLa and 293T cells. LINC03057 (also known as lnc-HOXB8-1:2 or RP11–357H14.17) is a lncRNA associated with the progression of different tumors, such as colorectal, gastric and endometrial carcinoma, with shortened overall survival and poor prognosis. Its high expression was associated with invasion depth, increased tumor size, lymphatic metastasis, and TNM (Tumor, Node, Metastasis) stage. LINC03057 acts as an miRNA sponge by negatively regulating hsa-miR-6825-5p, inducing tumor-associated macrophage infiltration, which promotes the progression of neuroendocrine differentiated colorectal cancer [18]. Moreover, LINC03057 plays an oncogene role in activating ATF2 (activating transcription factor 2) signaling and enhancing Treg cells, thus promoting cell proliferation, migration, and invasion in diffused gastric cancer [42,43,44]. This lncRNA, along with a few other lncRNAs, has also been reported to influence immune responses by modulating cellular migration and adhesion, as well as immune cell infiltration in the head and neck squamous cell carcinoma microenvironment [45].

cREL is one of the five members of the NF-κB family, which share a highly conserved DNA binding domain. In particular, c-Rel belongs to the NF-kB class II proteins, containing an additional transcription activation domain responsible for recruiting co-activators. In general, it has been described as a transcriptional activator that is able to determine a permissive chromatin environment at regulated gene promoters [46]. C-Rel regulates different cellular functions, including the expression of the antiapoptotic gene Bcl-xL [47] and a component of the checkpoint kinase Chk1 signaling pathway in an osteosarcoma cell line [48]. The role of the c-Rel subunit has been reported in human diseases, such as cardiac hypertrophy, fibrosis, and inflammatory bowel disease; its dysregulation has been observed in both liquid and solid tumors with an oncogenic or tumor suppressor function [27,49].

MALAT1, also known as LINC00047 (ENSG00000251562), is one of the most studied lncRNAs. It is highly conserved and abundantly expressed in cells and tissues but predominantly located in the nucleus. It is involved in numerous cellular processes, such as cell proliferation, regulation of gene expression, both at the transcriptional and post-transcriptional levels, RNA processing, epigenetic control, and nuclear organization [50,51]. MALAT1 affects various miRNAs, acting as endogenous RNA sponges, consequently increasing the gene expression of several essential genes involved in cancer progression and metastasis [52,53]. Recent research has demonstrated that MALAT-1 plays a role in mediating the EMT, which leads to the acquisition of stem cell-like properties and chemoresistance by interacting with various intracellular signaling pathways, including PI3K/Akt/mTOR and Wnt/β-catenin [53,54]. Overexpression of MALAT1 was initially identified in non-small cell lung cancer (NSCLC) and has been correlated with tumor initiation, progression, distant metastasis, autophagy, drug resistance, and poor outcome in several types of tumor [52], such as glioblastoma [55], breast cancer [50], colorectal cancer [21], acute myeloid leukemia [56]. Accumulating evidence suggests that upregulation of MALAT1 affects various molecular pathways, thus playing an essential role in a wide range of other diseases, including renin–angiotensin–aldosterone system, which is involved in blood pressure regulation and cardiovascular diseases [57], insulin signaling, type 2 diabetes [51], liver [58] and kidney disease [59]. Differential expression of MALAT1 has been reported under various physiological stresses such as serum starvation and hypoxia [51].

Our study provides evidence that miR-34a is required to regulate the expression of MALAT1. Through miR-34a ablation in two cell lines, RNA seq and qPCR, we demonstrated that the absence of miR-34a affects MALAT1 expression. Knockdown of endogenous miR-34a-5p in A375 melanoma cells resulted in an increase in MALAT1 levels, further confirming the regulatory relationship. These findings align with previous studies indicating that miR-34a functions as a tumor suppressor by targeting key oncogenic pathways [4]. The inverse relationship between miR-34a and MALAT1, consistent with a role for MALAT1 as an inhibitor of miR-34a activity, was described in melanoma and osteosarcoma [28,60,61] and was also reported with aging in mouse skeletal muscle [62]. A specific response element for miR-34a in the MALAT1 transcripts has been previously identified by bioinformatics analysis [34]. Furthermore, it has been demonstrated that, by miR-34a sponge activity, MALAT1 regulates cMyc and Met expression [28].

The biological implications of this regulation are particularly relevant in the biology of cancer, as both miR-34a and MALAT1 ncRNAs have been implicated in tumor progression. MiR-34a inhibits cell proliferation and promotes apoptosis, while MALAT1 is linked with enhanced metastatic potential and resistance to therapy. The apparent inverse correlation observed between their expression levels in some cancerous tissues [63,64,65,66,67] supports the thesis that miR-34a may exert its tumor-suppressive function, at least in part, through MALAT1 downregulation. In this respect, MALAT1 upregulation in breast cancer patients and cell lines has been recently correlated to low innate and adaptive immune response due, at least partially, to the interaction with miR-34a target. This interaction alters the expression of miR-34a and miR-17–5p and modulates MICA//MICB (MHC class I-related chain A and B), PDL1 (programmed death ligand-1), and B7-H4 (member of B7 family) expression on triple-negative breast cancer cells [68].

**Figure 5:**
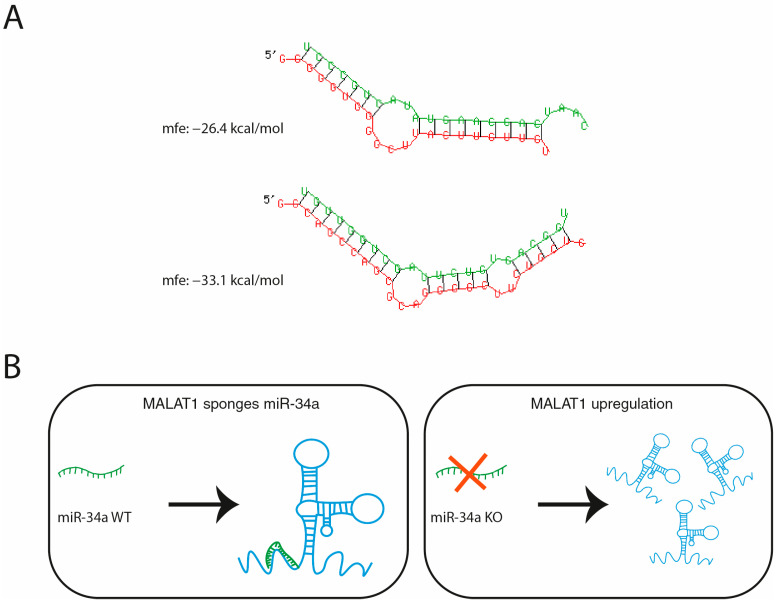
Proposed mechanism of miR-34a/MALAT1 interaction. (**A**) Structural prediction realized with RNAhybrid 2.2 of the possible interaction between miR-34a-3p (upper part, in green) and miR-34a-5p (lower part, in green) with MALAT1 (in red). The minimum free energy (mfe) structure for both interactions is shown and the mfe value is reported. (**B**) Proposed molecular model in which miR-34a is normally sponged by MALAT1 lncRNA (on the left), while in miR-34a KO cell lines the absence of miR-34a leads to increased MALAT1 expression (on the right, red cross).

The results of our study are in agreement with previously published results that showed MALAT1 regulation by miRNA interaction. A previous study has provided evidence that miR-9 targets MALAT1, binding two different miRNA binding sites and leading to the degradation of MALAT1 [69]. A similar mechanism may be envisaged for the action of miR-34a, based on a predicted binding site for miR34a in MALAT1 by in silico analyses (Figure 5A). We have schematized the possible mechanisms of reciprocal actions of MALTA1 as a sponge of miR-34a and miR-34a action on the expression of MALAT1 (Figure 5B).

Some limitations of this study must be acknowledged. We have chosen to compare the KO cell lines to parental WT lines as described in the literature. [70,71,72,73,74]. However, to rule out the negative effect by CRSPR/Cas9 procedure, we are aware that additional appropriate negative controls can be represented by additional clonal cell lines selected by puromycin after Cas9 transfection with gRNAs targeting a safe harbor gene, such as AAVS1 (Adeno-Associated Virus Integration Site 1) [75,76,77]. Furthermore, the precise molecular mechanism through which miR-34a may influence MALAT1 expression needs to be fully elucidated. Additionally, our study primarily focused on in vitro cell models using only one clone for both HeLa and 293T cells and further validations in primary cell lines, and in in vivo systems are required to confirm the molecular relevance of these interactions. Finally, given the pleiotropic role of both miR-34a and MALAT1, it will be essential to investigate their cross-talk in different molecular pathways to better understand their functional roles.

## 4. Materials and Methods

### 4.1. Cell Cultures

Human cervical cancer cell line HeLa, human embryonic kidney cell line 293T, and human melanoma cell line A375 were cultured in Dulbecco’s Modified Eagle Medium (DMEM), containing 10% of Fetal Bovine Serum (FBS) and 1% of sodium pyruvate, L–Glutamine, penicillin, and streptomycin. All cell cultures were maintained in a humidified incubator at 37 °C with 5% CO_2_.

### 4.2. miR-34a Knockout Cell Line Production

To produce HeLa and 293T miR-34a KO cell lines, three guide RNAs (gRNAs) were designed using the online tool CHOPCHOP v3, previously described [17,78,79]. The three gRNAs were selected for their high target specificity (low off-target site recognition) and their location on the target sequence, the pre-miR-34a sequence (Figure S1 and Table S1). The plasmid expressing pSpCas9 and each gRNA was prepared by cloning each gRNA independently into BbsI restriction sites of the pSpCas9(BB)-2A-Puro (PX459) V2.0 vector (#62988, Addgene, Watertown, MA, USA) using the T4 DNA ligase (Promega Corporation, Madison, WI, USA). Sequences of the gRNAs are reported in Table 1. HeLa and 293T cells were transfected using the Trans-IT^®^-LT1 transfection reagent (MIR2300, Mirus Bio, Madison, WI, USA), following the manufacturer’s protocol. To produce the miR-34a-KO cell lines, HeLa and 293T were seeded at a concentration of 2.5 × 10^5^ cells/well and 4 × 10^5^ cells/well, respectively, in 6-well plates. After 20 h (70–80% confluence), the culture medium was removed and replaced with an antibiotic-deficient medium. Cells were subsequently transfected with the pSpCas9(BB)-2A (PX459) V2.0 plasmid vector (Addgene–Plasmid #62988, Addgene, Watertown, MA, USA), containing the CRISPR/Cas9 system and the gRNAs, using 2 µL of TransIT-LT1 transfection reagent (Mirus Bio, Madison, WI, USA) for each µg of DNA. After the addition of TransIT-LT1 transfection agent, the cells were maintained in an incubator and selected in 0,5 μg/mL puromycin. Clonal cell lines were separated by limiting dilution using discrete Poisson distribution probability.

### 4.3. miR-34a Knock-Out Validation and miR-34b and miR-34c Expression Analysis

Effectiveness of the knockout approach was evaluated via reverse transcription–quantitative PCR (RT-qPCR) using the TaqMan MicroRNA Assay kit (Applied Biosystems, Waltham, MA, USA) with probes for hsa-miR-34a (Applied Biosystems, Waltham, MA, USA, assay ID 000426), hsa-miR-34b (Applied Biosystems, Waltham, MA, USA, assay ID 00427), hsa-miR-34c (Applied Biosystems, Waltham, MA, USA, assay ID 00428) and the internal reference small RNA U6 (Applied Biosystems, Waltham, MA, USA, assay ID 001973) for HeLa and 293T cells and miR-191-5p (Applied Biosystems, Waltham, MA, USA, assay ID 002299) for A375 melanoma cells, according to manufacturer’s instructions. Expression levels of miR-34a in the KO clones were analyzed using the 2^−ΔΔCt^ method [80] and normalized for U6 or miR-191-5p expression. Additionally, Sanger sequencing of the edited region was used to further confirm the presence of deletions in the desired gene in the miR-34a KO cell lines. The sequences were aligned using Clustal Omega version 1.2.4 [81].

### 4.4. Cell Proliferation Analysis

HeLa and 293T cells were seeded in 96-well plates at a starting concentration of 1 × 10^3^ cells/well and 1 × 10^4^ cells/well, respectively. After 24 h, 10 µL of water-soluble tetrazolium salt reagent (Cell Counting Kit 8, Dojindo Molecular Technologies, Munich, Germany) was added to each well. After two hours, cell proliferation at the starting timepoint (T0) was evaluated by measuring absorbance at 450 nm via a spectrophotometer (Byo-noy 96 plate reader, Enzo Life Sciences, Bruxelles, Belgium). The same procedure was repeated at different timepoints (24, 48 and 72 h). Absorbance from wells containing only the medium and the tetrazolium salt reagents was used as a blank. Proliferation was measured as proliferative rate compared to T0. The cell proliferation analysis was performed at least three times on each cell line, using three technical replicates for each timepoint.

### 4.5. RNA Extraction for Next-Generation Sequencing (NGS) of HeLa WT and miR-34a KO Cells

Total RNA was extracted from 3 WT samples and 3 miR-34a KO HeLa cell clones by TRIzol (ThermoFisher Scientific, Waltham, MA, USA), according to the manufacturer’s instructions and under RNase-free conditions. Quality control of the 6 extracted RNAs included the evaluation of the yield, purity, and integrity. RNA quantity was assessed using both the NanoDrop ND-100 spectrophotometer (ThermoFisher Scientific, Waltham, MA, USA) and the Invitrogen Qubit 2.0 fluorometer (ThermoFisher Scientific, Waltham, MA, USA).

RNA purity was determined by measuring the 260/280 and 260/230 nm absorbance ratios (higher than 1.8 and between 2.0 and 2.2, respectively), as evaluated using the NanoDrop ND-100 spectrophotometer (ThermoFisher Scientific, Waltham, MA, USA). RNA integrity was determined by capillary electrophoresis using Fragment Analyzer (Agilent Technologies, Santa Clara, CA, USA), yielding an RQN (RNA Quality Number) greater than 7.9 for each RNA sample.

### 4.6. Library Preparation and RNA-Sq of HeLa WT and miR-34a KO Cells

Total RNA samples were sent to BMR Genomics,(Padua, Italy), for the Transcriptomic analyses. According to the Company’s procedures, RNA samples were paired-end sequenced on NovaSeq 6000 (Illumina, San Diego, CA, USA) with a read length of 2 × 100 bp and a mean read depth of 2 × 20 million reads per library. Briefly, starting with 1 µg of total RNA per sample, mRNA was polyA-selected and fragmented. The TruSeq Stranded mRNA Library Preparation kit (Illumina, San Diego, CA, USA) was used to prepare first-strand cDNAs by random hexamer priming and to synthesize second-strand cDNAs ready for library construction. The cDNAs were end-repaired and adenylated before being ligated with multiple indexed adapters and were then PCR-enriched and purified to create the final 6 cDNA libraries. After a quality check of the libraries using Fragment Analyzer (Agilent Technologies, Santa Clara, CA, USA), the libraries were normalized, pooled, and sequenced by the NovaSeq 6000 flow cell.

The RNAseq raw data have been made available through the European Nucleotide Archive (ENA) portal (accession PRJEB91112).

### 4.7. Data Processing for RNA-Seq of HeLa WT and miR-34a KO Cells

The bioinformatic analysis of the data obtained by RNA-seq was performed by BMR Genomics, Padua, Italy. Data processing protocols applied by the company are described below.

Raw paired-end sequencing reads obtained by the Illumina system were pre-processed using fastp v0.20.0 [82], applying parameters to remove residual adapter sequences and keep only high-quality data.

Passing filter reads were mapped and aligned to the genome reference (*Homo sapiens*) using STAR v2.7.9.a [83] with standard parameters, except for the sjdbOverhang option set on read length. Genome and transcript annotations provided as input were downloaded from v105 of the Ensembl repository. Alignments were then elaborated by RSEM v1.3.3 [84] to estimate transcript and gene abundances.

Differential expression was computed by edgeR [85] from raw counts in each comparison. Multiple testing controlling procedure was applied, and genes with a *p*-value ≤ 0.05 and logFC > |0.5| were considered differentially expressed. Annotation of differentially expressed genes was performed using the BioMart package [86] inR 4.3, querying available Ensembl Gene IDs and retrieving Gene Names and Entrez gene IDs.

The heatmap represents a selection of the 50 most differentially expressed genes based on the logFC value. A Volcano Plot representative of the RNA seq was produced using SRplot [87]. Gene ontology (GO)/Kyoto Encyclopedia of Genes and Genomes (KEGG v3) enrichment analyses were performed using the GO pathway enrichment bubble plot tool from SRplot [87], accessed on 17 April 2025.

### 4.8. miR-34a Phenotypic Rescue in 293T KO Cell Line

293T miR-34a KO cells were seeded in 6-well plates at a starting concentration of 4 × 10^5^ cells/well. After 24 h, cells were transfected with pSG5-miR-34a plasmid or pSG5 empty vector as control, kindly gifted from Martin Hart’s Laboratory [88], using the Trans-IT^®^-LT1 transfection reagent (MIR2300, Mirus Bio, Madison, WI, USA) and according to the manufacturer’s protocol. RNA was extracted and analyzed as described in Section 4.10. Efficient miR-34a overexpression was evaluated as described in Section 4.3.

### 4.9. miR-34a Inhibition in A375 Cell Line

A375 cells were seeded in 6-well plates at a concentration of 3 × 10^5^ cells/well and transfected with miRNA Inhibitor Negative Control (Cohesion Biosciences, Cat. CIH0000-5NMOL, London, UK) and hsa-miR-34a-5p miRNA Inhibitor (Cohesion Biosciences, Cat. CIH0060-10NMOL, London, UK), using the Lipofectamine™ 3000 Transfection Reagent (Invitrogen, Cat L3000001, Waltham, MA, USA), following the protocol’s instructions. After 48 h, RNA was extracted and analyzed as described in Section 4.10. Efficient miR-34a-5p inhibition was evaluated as described in Section 4.3.

### 4.10. RNA Isolation and RT-qPCR

Total RNA was extracted from cells using Qiazol reagent (Qiagen, Venlo, NL, USA) followed by phase separation employing chloroform. Following RNA precipitation by isopropanol/ethanol, the pellet was resuspended using nuclease-free water and quantified by Nanodrop 1000 V3.7.1 Spectrophotometer (ThermoFisher Scientific, Waltham, MA, USA). One microgram of total RNA was subjected to first-strand cDNA synthesis using QuantiTect Reverse Transcription kit (Qiagen, Venlo, NL). The cDNA was used to assess gene expression via RT-qPCR using the SensiFAST^TM^ SYBR^®^ No-Rox kit (Meridian Biosciences, Cincinnati, OH, USA). The primers, relative to the sequences of REL (REL proto-oncogene, NF-κB subunit), MAP1B (Microtubule-Associated Protein 1B), ONECUT2 (One Cut Homeobox 2), MALAT1 (Metastasis Associated Lung Adenocarcinoma Transcript 1), LINC03057 (Long Intergenic Non-Protein Coding RNA 3057), and RPLP0 (Ribosomal Protein Lateral Stalk Subunit P0) genes used for the quantitative analyses, are reported in Table 2. RPLP0 was used as an internal reference gene. Gene expression was analyzed using the 2^−ΔΔCt^ method [80] and normalized for RPLP0 expression.

### 4.11. Bioinformatic Prediction of miR-34a/MALAT1 Interaction

Structural prediction of the possible interaction between MALAT1 and miR-34a-3p or miR-34a-5p was performed using RNAhybrid version 2.2 [89]. For MALAT1, a reference sequence of 8779 nucleotides of length was used as input (NR_002819.4), while sequences for miR-34a-5p and miR-34a-3p were retrieved from miRbase (accession MIMAT0000255 and MIMAT0004557, respectively). RNAhybrid was set to produce only the best possible interaction between the lncRNA and the miRNA, retrieving only the structure showing the lowest minimum free energy.

### 4.12. Statistical Analysis

Data are expressed as mean ± SD. Statistical differences were calculated by two-tailed Student’s t-test or one-way ANOVA test using GraphPad Prism v8 (GraphPad Inc., San Diego, CA, USA), where *p* ≤ 0.05 was considered statistically significant.

## 5. Conclusions

This study shows that knockout or inhibition of miR-34a non-coding RNA deregulates the expression of LINC03057, REL, and MALAT1 genes. Specifically, the absence or reduced expression of miR-34a leads to increased expression of MALAT1 in three different cell types. Our findings provide novel insights into the regulatory network modulating MALAT1 expression and suggest that targeting the miR-34a/MALAT1 axis could represent a potential therapeutic approach in diseases where MALAT1 is dysregulated. Future studies should explore these interactions and their effect on pathway-specific cellular responses.

## Figures and Tables

**Table 1:** Target and gRNA sequences.

gRNA	Target Sequence	gRNA Sequence (5′-3′)
gRNA 1	TTCTTTGGCAGTGTCTTAGCTGG	Fw: CACCGTTCTTTGGCAGTGTCTTAGC Rv: AAACGCTAAGACACTGCCAAAGAAC
gRNA 2	GCCAGCTGTGAGTGTTTCTTTGG	Fw: CACCGCCAGCTGTGAGTGTTTCTT Rv: AAACAAGAAACACTCACAGCTGGC
gRNA 3	TAGAAGTGCTGCACGTTGTGGGG	Fw: CACCGCACAACGTGCAGCACTTCTA Rv: AAACTAGAAGTGCTGCACGTTGTGC

**Table 2:** Primers used for RT-qPCR.

Target	Primer Sequence (5’-3’)
REL	Fw: ATTTGACGACTGCTCTTCCTC Rv: TCCTCTGACACTTCCACAATTC
MAP1B	Fw: CTCCTTCCAGAACTTCATAGAGATT Rv: TTCAGGACAGAACAGGGTTAAG
ONECUT2	Fw: GGAATCCAAAACCGTGGAGTAA Rv: CTCTTTGCGTTTGCACGCTG
MALAT1	Fw: ATGCGAGTTGTTCTCCGTCT Rv: TATCTGCGGTTTCCTCAAGC
LINC03057	Fw: TGTTCTGCGTCTGTGTCTAC Rv: CCACTCCCTTTCTTCCTTGAA
RPLP0	Fw: ACATGTTGCTGGCCAATAAGGT Rv: CCTAAAGCCTGGAAAAAGGAGG

## Data Availability

Raw reads produced from RNA-seq experiments are available at the European Nucleotide Archive (ENA) browser under the accession number PRJEB91112. Excel datasheets containing raw and elaborated data from Figure 1, Figure 2, Figure 3 and Figure 4 and statistically significant differentially expressed genes from Figure S3 are available on Zenodo (https://doi.org/10.5281/zenodo.15913985).

## Supplementary Materials

The following supporting information can be downloaded at https://www.mdpi.com/article/10.3390/ncrna11040060/s1, Figure S1—gRNA design and strategy for miR-34a KO generation; Figure S2—Alignment of Sanger sequences from WT and miR-34a KO cells and PCR verification of the presence of deletion in the KO cells; Figure S3—NGS RNA-seq analyses in HeLa WT and miR-34a KO cells; Figure S4—Gene Ontology and KEGG analysis of RNA-seq data; Table S1—CHOPCHOP prediction of off-target effects for the three guide RNAs used.

## Abbreviations

The following abbreviations are used in this manuscript:

3’-UTR	3’-Untranslated Region
Akt	AKT Serine/Threonine kinase
ANOVA	Analysis of Variance
ATF2	Activating Transcription Factor 2
BCL2	B-cell lymphoma 2
Bcl-xL	B-cell lymphoma extra large
CCK8	Cell Counting Kit 8
CCND1	Cyclin D1
CDK4	Cyclin Dependent Kinase 4
CDK6	Cyclin Dependent Kinase 6
cDNA	Complementary DNA
Chk1	Checkpoint Kinase 1
c-Myc	MYC proto-oncogene
CRISPR-Cas9	Clustered Regularly Interspaced Short Palindromic Repeats Cas9 associated
DMEM	Dulbecco’s Modified Eagle Medium
EMT	Epithelial–Mesenchymal transition
FBS	Fetal Bovine Serum
FC	Fold Change
GO	Gene Ontology
gRNA	Guide RNA
KEGG	Kyoto Encyclopedia of Genes and Genomes
KO	Knock–out
LINC03057	Long Intergenic Non-Protein Coding RNA 3057
lncRNA	Long non-coding RNA
MALAT1	Metastasis Associated Lung Adenocarcinoma Transcript 1
MAP1B	Microtubule-Associated Protein 1B
Met	MET proto-oncogene
miRNA	Micro-RNA
mfe	Minimum Free Energy
mTOR	Mechanistic Target Of Rapamycin Kinase
NGS	Next-Generation Sequencing
NSCLC	Non-small cell lung cancer
ONECUT2	One Cut Homeobox 2
PI3K	Phosphatidylinositol 3-kinase
qPCR	Quantitative PCR
REL	REL proto-oncogene, NF-κB subunit
RNA-seq	RNA sequencing
RPLP0	Ribosomal Protein Lateral Stalk Subunit P0
RQN	RNA quality number
RT-qPCR	Reverse transcription–quantitative PCR
SD	Standard Deviation
SIRT1	Sirtuin 1
TNM	Tumor, Node, Metastasis
Wnt	Wnt Family Member 1
WT	Wild-Type

## References

[1] Peng Y., Croce C.M. (2016). The Role of MicroRNAs in Human Cancer. Signal Transduct. Target. Ther..

[2] Svoronos A.A., Engelman D.M., Slack F.J. (2016). OncomiR or Tumor Suppressor? The Duplicity of MicroRNAs in Cancer. Cancer Res..

[3] Ali Syeda Z., Langden S.S.S., Munkhzul C., Lee M., Song S.J. (2020). Regulatory Mechanism of MicroRNA Expression in Cancer. Int. J. Mol. Sci..

[4] Li W.J., Wang Y., Liu R., Kasinski A.L., Shen H., Slack F.J., Tang D.G. (2021). MicroRNA-34a: Potent Tumor Suppressor, Cancer Stem Cell Inhibitor, and Potential Anticancer Therapeutic. Front. Cell Dev. Biol..

[5] Bader A.G. (2012). miR-34—A microRNA Replacement Therapy Is Headed to the Clinic. Front. Genet..

[6] Rokavec M., Li H., Jiang L., Hermeking H. (2014). The P53/miR-34 Axis in Development and Disease. J. Mol. Cell Biol..

[7] Ji Q., Hao X., Meng Y., Zhang M., Desano J., Fan D., Xu L. (2008). Restoration of Tumor Suppressor miR-34 Inhibits Human P53-Mutant Gastric Cancer Tumorspheres. BMC Cancer.

[8] Sun F., Fu H., Liu Q., Tie Y., Zhu J., Xing R., Sun Z., Zheng X. (2008). Downregulation of CCND1 and CDK6 by miR-34a Induces Cell Cycle Arrest. FEBS Lett..

[9] Fu J., Imani S., Wu M.-Y., Wu R.-C. (2023). MicroRNA-34 Family in Cancers: Role, Mechanism, and Therapeutic Potential. Cancers.

[10] Hermeking H. (2012). MicroRNAs in the P53 Network: Micromanagement of Tumour Suppression. Nat. Rev. Cancer.

[11] Mazziotta C., Cervellera C.F., Lanzillotti C., Touzé A., Gaboriaud P., Tognon M., Martini F., Rotondo J.C. (2023). MicroRNA Dysregulations in Merkel Cell Carcinoma: Molecular Mechanisms and Clinical Applications. J. Med. Virol..

[12] Siemens H., Jackstadt R., Hünten S., Kaller M., Menssen A., Götz U., Hermeking H. (2011). miR-34 and SNAIL Form a Double-Negative Feedback Loop to Regulate Epithelial-Mesenchymal Transitions. Cell Cycle.

[13] Raver-Shapira N., Marciano E., Meiri E., Spector Y., Rosenfeld N., Moskovits N., Bentwich Z., Oren M. (2007). Transcriptional Activation of miR-34a Contributes to P53-Mediated Apoptosis. Mol. Cell.

[14] Chang T.-C., Wentzel E.A., Kent O.A., Ramachandran K., Mullendore M., Lee K.H., Feldmann G., Yamakuchi M., Ferlito M., Lowenstein C.J. (2007). Transactivation of miR-34a by P53 Broadly Influences Gene Expression and Promotes Apoptosis. Mol. Cell.

[15] Tarasov V., Jung P., Verdoodt B., Lodygin D., Epanchintsev A., Menssen A., Meister G., Hermeking H. (2007). Differential Regulation of microRNAs by P53 Revealed by Massively Parallel Sequencing: miR-34a Is a P53 Target That Induces Apoptosis and G1-Arrest. Cell Cycle.

[16] Bommer G.T., Gerin I., Feng Y., Kaczorowski A.J., Kuick R., Love R.E., Zhai Y., Giordano T.J., Qin Z.S., Moore B.B. (2007). P53-Mediated Activation of miRNA34 Candidate Tumor-Suppressor Genes. Curr. Biol..

[17] Labun K., Montague T.G., Krause M., Torres Cleuren Y.N., Tjeldnes H., Valen E. (2019). CHOPCHOP v3: Expanding the CRISPR Web Toolbox beyond Genome Editing. Nucleic Acids Res..

[18] Li X., Lan Q., Lai W., Wu H., Xu H., Fang K., Chu Z., Zeng Y. (2022). Exosome-Derived Lnc-HOXB8-1:2 Induces Tumor-Associated Macrophage Infiltration to Promote Neuroendocrine Differentiated Colorectal Cancer Progression by Sponging Hsa-miR-6825-5p. BMC Cancer.

[19] Youness R.A., Khater N., El-Khouly A., Nafea H., Manie T., Habashy D., Gad M.Z. (2025). Direct and Indirect Modulation of STAT3/CSE/H2S Axis in Triple Negative Breast Cancer by Non-Coding RNAs: MALAT-1 lncRNA, miR-486-5p and miR-30a-5p. Pathol. Res. Pract..

[20] Bai X., Li J., Guo X., Huang Y., Xu X., Tan A., Jia Y., Sun Q., Guo X., Chen J. (2024). LncRNA MALAT1 Promotes Erastin-Induced Ferroptosis in the HBV-Infected Diffuse Large B-Cell Lymphoma. Cell Death Dis..

[21] Masrour M., Khanmohammadi S., Habibzadeh A., Fallahtafti P. (2024). LncRNA MALAT1 as Diagnostic and Prognostic Biomarker in Colorectal Cancers: A Systematic Review and Meta-Analysis. PLoS ONE.

[22] Ning J., Yang R., Wang H., Ma H., Cui L. (2024). LncRNA MALAT1 Silencing Represses CXCL12-Induced Proliferation, Invasion, and Homing Behavior in Multiple Myeloma by Inhibiting CXCR4. Hematology.

[23] Luo J., Hu Q., Gou M., Liu X., Qin Y., Zhu J., Cai C., Tian T., Tu Z., Du Y. (2021). Expression of Microtubule-Associated Proteins in Relation to Prognosis and Efficacy of Immunotherapy in Non-Small Cell Lung Cancer. Front. Oncol..

[24] Chien T.-M., Chan T.-C., Huang S.K.-H., Yeh B.-W., Li W.-M., Huang C.-N., Li C.-C., Wu W.-J., Li C.-F. (2020). Role of Microtubule-Associated Protein 1b in Urothelial Carcinoma: Overexpression Predicts Poor Prognosis. Cancers.

[25] Inoue H., Kanda T., Hayashi G., Munenaga R., Yoshida M., Hasegawa K., Miyagawa T., Kurumada Y., Hasegawa J., Wada T. (2024). A MAP1B–Cortactin–Tks5 Axis Regulates TNBC Invasion and Tumorigenesis. J. Cell Biol..

[26] Yu J., Li D., Jiang H. (2020). Emerging Role of ONECUT2 in Tumors. Oncol. Lett..

[27] Hunter J.E., Leslie J., Perkins N.D. (2016). C-Rel and Its Many Roles in Cancer: An Old Story with New Twists. Br. J. Cancer.

[28] Li F., Li X., Qiao L., Liu W., Xu C., Wang X. (2019). MALAT1 Regulates miR-34a Expression in Melanoma Cells. Cell Death Dis..

[29] Zhang L., Liao Y., Tang L. (2019). MicroRNA-34 Family: A Potential Tumor Suppressor and Therapeutic Candidate in Cancer. J. Exp. Clin. Cancer Res..

[30] Ding R., Su D., Zhao Q., Wang Y., Wang J.-Y., Lv S., Ji X. (2023). The Role of microRNAs in Depression. Front. Pharmacol..

[31] Bavamian S., Mellios N., Lalonde J., Fass D.M., Wang J., Sheridan S.D., Madison J.M., Zhou F., Rueckert E.H., Barker D. (2015). Dysregulation of miR-34a Links Neuronal Development to Genetic Risk Factors for Bipolar Disorder. Mol. Psychiatry.

[32] Andolina D., Di Segni M., Accoto A., Lo Iacono L., Borreca A., Ielpo D., Berretta N., Perlas E., Puglisi-Allegra S., Ventura R. (2018). MicroRNA-34 Contributes to the Stress-Related Behavior and Affects 5-HT Prefrontal/GABA Amygdalar System through Regulation of Corticotropin-Releasing Factor Receptor 1. Mol. Neurobiol..

[33] Chen B.-Y., Lin J.-J., Lu M.-K., Tan H.-P., Jang F.-L., Lin S.-H. (2021). Neurodevelopment Regulators miR-137 and miR-34 Family as Biomarkers for Early and Adult Onset Schizophrenia. npj Schizophr..

[34] Chua C.E.L., Tang B.L. (2019). miR-34a in Neurophysiology and Neuropathology. J. Mol. Neurosci..

[35] Hu Y., Pei W., Hu Y., Li P., Sun C., Du J., Zhang Y., Miao F., Zhang A., Shen Y. (2020). MiR34a Regulates Neuronal MHC Class I Molecules and Promotes Primary Hippocampal Neuron Dendritic Growth and Branching. Front. Cell. Neurosci..

[36] Bernardo B.C., Gao X.-M., Tham Y.K., Kiriazis H., Winbanks C.E., Ooi J.Y.Y., Boey E.J.H., Obad S., Kauppinen S., Gregorevic P. (2014). Silencing of miR-34a Attenuates Cardiac Dysfunction in a Setting of Moderate, but Not Severe, Hypertrophic Cardiomyopathy. PLoS ONE.

[37] Zhang X.-L., Zhang G., Bai Z.-H. (2021). miR-34a Attenuates Myocardial Fibrosis in Diabetic Cardiomyopathy Mice via Targeting Pin-1. Cell Biol. Int..

[38] Ni T., Lin N., Lu W., Sun Z., Lin H., Chi J., Guo H. (2020). Dihydromyricetin Prevents Diabetic Cardiomyopathy via miR-34a Suppression by Activating Autophagy. Cardiovasc. Drugs Ther..

[39] Boon R.A., Iekushi K., Lechner S., Seeger T., Fischer A., Heydt S., Kaluza D., Tréguer K., Carmona G., Bonauer A. (2013). MicroRNA-34a Regulates Cardiac Ageing and Function. Nature.

[40] Fochi S., Giuriato G., De Simone T., Gomez-Lira M., Tamburin S., Del Piccolo L., Schena F., Venturelli M., Romanelli M.G. (2020). Regulation of microRNAs in Satellite Cell Renewal, Muscle Function, Sarcopenia and the Role of Exercise. Int. J. Mol. Sci..

[41] Hua C.-C., Liu X.-M., Liang L.-R., Wang L.-F., Zhong J.-C. (2021). Targeting the microRNA-34a as a Novel Therapeutic Strategy for Cardiovascular Diseases. Front. Cardiovasc. Med..

[42] Yang B., Luo T., Zhang M., Lu Z., Xue X., Fang G. (2017). The Novel Long Noncoding RNA RP11-357H14.17 Acts as an Oncogene by Promoting Cell Proliferation and Invasion in Diffuse-Type Gastric Cancer. OncoTargets Ther..

[43] Xiaoli T., Wenting W., Meixiang Z., Chunlei Z., Chengxia H. (2021). Long Noncoding RNA RP11-357H14.17 Plays an Oncogene Role in Gastric Cancer by Activating ATF2 Signaling and Enhancing Treg Cells. Biomed. Res. Int..

[44] Liu T., Ma Y., Han S., Sun P. (2024). Genome-Wide Investigation of lncRNAs Revealed Their Tight Association with Gastric Cancer. J. Cancer Res. Clin. Oncol..

[45] Lan T., Yan Y., Zheng D., Ding L. (2024). Investigating Diagnostic Potential of Long Non-Coding RNAs in Head and Neck Squamous Cell Carcinoma Using TCGA Database and Clinical Specimens. Sci. Rep..

[46] van Essen D., Zhu Y., Saccani S. (2010). A Feed-Forward Circuit Controlling Inducible NF-κB Target Gene Activation by Promoter Histone Demethylation. Mol. Cell.

[47] Gilmore T.D., Gerondakis S. (2011). The C-Rel Transcription Factor in Development and Disease. Genes Cancer.

[48] Kenneth N.S., Mudie S., Rocha S. (2010). IKK and NF-kappaB-Mediated Regulation of Claspin Impacts on ATR Checkpoint Function. EMBO J..

[49] Leslie J., Hunter J.E., Collins A., Rushton A., Russell L.G., Ramon-Gil E., Laszczewska M., McCain M., Zaki M.Y.W., Knox A. (2023). C-Rel-Dependent Chk2 Signaling Regulates the DNA Damage Response Limiting Hepatocarcinogenesis. Hepatology.

[50] Thapa R., Afzal O., Gupta G., Bhat A.A., Almalki W.H., Alzarea S.I., Kazmi I., Altamimi A.S.A., Subramaniyan V., Thangavelu L. (2023). Unveiling the Connection: Long-Chain Non-Coding RNAs and Critical Signaling Pathways in Breast Cancer. Pathol. Res. Pract..

[51] Arun G., Aggarwal D., Spector D.L. (2020). MALAT1 Long Non-Coding RNA: Functional Implications. Noncoding RNA.

[52] Mazarei M., Shahabi Rabori V., Ghasemi N., Salehi M., Rayatpisheh N., Jahangiri N., Saberiyan M. (2023). LncRNA MALAT1 Signaling Pathway and Clinical Applications in Overcome on Cancers Metastasis. Clin. Exp. Med..

[53] Hussein M.A., Valinezhad K., Adel E., Munirathinam G. (2024). MALAT-1 Is a Key Regulator of Epithelial-Mesenchymal Transition in Cancer: A Potential Therapeutic Target for Metastasis. Cancers.

[54] Hussain M.S., Altamimi A.S.A., Afzal M., Almalki W.H., Kazmi I., Alzarea S.I., Saleem S., Prasher P., Oliver B., Singh S.K. (2024). From Carcinogenesis to Therapeutic Avenues: lncRNAs and mTOR Crosstalk in Lung Cancer. Pathol. Res. Pract..

[55] Bagheri-Mohammadi S., Karamivandishi A., Mahdavi S.A., Siahposht-Khachaki A. (2024). New Sights on Long Non-Coding RNAs in Glioblastoma: A Review of Molecular Mechanism. Heliyon.

[56] Priya, Garg M., Talwar R., Bharadwaj M., Ruwali M., Pandey A.K. (2024). Clinical Relevance of Long Non-Coding RNA in Acute Myeloid Leukemia: A Systematic Review with Meta-Analysis. Leuk. Res..

[57] Sudhakaran G. (2025). Interplay between lncRNAs and microrNAs in Hypertension. Hypertens. Res..

[58] Ismail M., Fadul M.M., Taha R., Siddig O., Elhafiz M., Yousef B.A., Jiang Z., Zhang L., Sun L. (2024). Dynamic Role of Exosomal Long Non-Coding RNA in Liver Diseases: Pathogenesis and Diagnostic Aspects. Hepatol. Int..

[59] Puri B., Majumder S., Gaikwad A.B. (2025). LncRNA MALAT1 as a Potential Diagnostic and Therapeutic Target in Kidney Diseases. Pathol. Res. Pract..

[60] Sun Z., Zhang T., Chen B. (2019). Long Non-Coding RNA Metastasis-Associated Lung Adenocarcinoma Transcript 1 (MALAT1) Promotes Proliferation and Metastasis of Osteosarcoma Cells by Targeting c-Met and SOX4 via miR-34a/c-5p and miR-449a/b. Med. Sci. Monit..

[61] Duan G., Zhang C., Xu C., Xu C., Zhang L., Zhang Y. (2019). Knockdown of MALAT1 Inhibits Osteosarcoma Progression via Regulating the miR-34a/Cyclin D1 Axis. Int. J. Oncol..

[62] Ruan L., Mendhe B., Parker E., Kent A., Isales C.M., Hill W.D., McGee-Lawrence M., Fulzele S., Hamrick M.W. (2021). Long Non-Coding RNA MALAT1 Is Depleted With Age in Skeletal Muscle in Vivo and MALAT1 Silencing Increases Expression of TGF-Β1 in Vitro. Front. Physiol..

[63] Wu L., Wang X., Guo Y. (2017). Long Non-Coding RNA MALAT1 Is Upregulated and Involved in Cell Proliferation, Migration and Apoptosis in Ovarian Cancer. Exp. Ther. Med..

[64] Zou A., Liu R., Wu X. (2016). Long Non-Coding RNA MALAT1 Is up-Regulated in Ovarian Cancer Tissue and Promotes SK-OV-3 Cell Proliferation and Invasion. Neoplasma.

[65] Ying L., Chen Q., Wang Y., Zhou Z., Huang Y., Qiu F. (2012). Upregulated MALAT-1 Contributes to Bladder Cancer Cell Migration by Inducing Epithelial-to-Mesenchymal Transition. Mol. Biosyst..

[66] Mattie M.D., Benz C.C., Bowers J., Sensinger K., Wong L., Scott G.K., Fedele V., Ginzinger D., Getts R., Haqq C. (2006). Optimized High-Throughput microRNA Expression Profiling Provides Novel Biomarker Assessment of Clinical Prostate and Breast Cancer Biopsies. Mol. Cancer.

[67] Vilming Elgaaen B., Olstad O.K., Haug K.B.F., Brusletto B., Sandvik L., Staff A.C., Gautvik K.M., Davidson B. (2014). Global miRNA Expression Analysis of Serous and Clear Cell Ovarian Carcinomas Identifies Differentially Expressed miRNAs Including miR-200c-3p as a Prognostic Marker. BMC Cancer.

[68] Mekky R.Y., Ragab M.F., Manie T., Attia A.A., Youness R.A. (2023). MALAT-1: Immunomodulatory lncRNA Hampering the Innate and the Adaptive Immune Arms in Triple Negative Breast Cancer. Transl. Oncol..

[69] Leucci E., Patella F., Waage J., Holmstrøm K., Lindow M., Porse B., Kauppinen S., Lund A.H. (2013). microRNA-9 Targets the Long Non-Coding RNA MALAT1 for Degradation in the Nucleus. Sci. Rep..

[70] Kim J.S., Kim E.J., Lee S., Tan X., Liu X., Park S., Kang K., Yoon J.-S., Ko Y.H., Kurie J.M. (2019). MiR-34a and miR-34b/c Have Distinct Effects on the Suppression of Lung Adenocarcinomas. Exp. Mol. Med..

[71] Olajossy B., Slominski A.T., Wolnicka-Glubisz A. (2025). Inhibition of the RIPK4 Enhances Suppression of Human Melanoma Growth through Vitamin D Signaling. Mol. Cell Endocrinol..

[72] Bai R., Zhang S., Gu X., You Y., Liu X. (2025). Establishment of a TRPV2 Knockout Human Embryonic Stem Cell Line (WAe009-A-1Y) Using Episomal Vector-Based CRISPR/Cas9. Stem Cell Res..

[73] Zhang M., Jiang W.I., Arkelius K., Swanson R.A., Ma D.K., Singhal N.S. (2025). PATJ Regulates Cell Stress Responses and Vascular Remodeling Post-Stroke. Redox Biol..

[74] Correia L., Shalygin A., Erbacher A., Zaisserer J., Gudermann T., Chubanov V. (2025). TRPM7 Underlies Cadmium Cytotoxicity in Pulmonary Cells. Arch. Toxicol..

[75] Gu J., Rollo B., Sumer H., Cromer B. (2022). Targeting the AAVS1 Site by CRISPR/Cas9 with an Inducible Transgene Cassette for the Neuronal Differentiation of Human Pluripotent Stem Cells. Methods Mol. Biol..

[76] Kelly J.J., Saee-Marand M., Nyström N.N., Evans M.M., Chen Y., Martinez F.M., Hamilton A.M., Ronald J.A. (2021). Safe Harbor-Targeted CRISPR-Cas9 Homology-Independent Targeted Integration for Multimodality Reporter Gene-Based Cell Tracking. Sci. Adv..

[77] Yada R.C., Ostrominski J.W., Tunc I., Hong S.G., Zou J., Dunbar C.E. (2017). CRISPR/Cas9-Based Safe-Harbor Gene Editing in Rhesus iPSCs. Curr. Protoc. Stem Cell Biol..

[78] Ran F.A., Hsu P.D., Wright J., Agarwala V., Scott D.A., Zhang F. (2013). Genome Engineering Using the CRISPR-Cas9 System. Nat. Protoc..

[79] Fochi S., Bergamo E., Serena M., Mutascio S., Journo C., Mahieux R., Ciminale V., Bertazzoni U., Zipeto D., Romanelli M.G. (2019). TRAF3 Is Required for NF-κB Pathway Activation Mediated by HTLV Tax Proteins. Front. Microbiol..

[80] Livak K.J., Schmittgen T.D. (2001). Analysis of Relative Gene Expression Data Using Real-Time Quantitative PCR and the 2(-Delta Delta C(T)) Method. Methods.

[81] Madeira F., Madhusoodanan N., Lee J., Eusebi A., Niewielska A., Tivey A.R.N., Lopez R., Butcher S. (2024). The EMBL-EBI Job Dispatcher Sequence Analysis Tools Framework in 2024. Nucleic Acids Res..

[82] Chen S., Zhou Y., Chen Y., Gu J. (2018). Fastp: An Ultra-Fast All-in-One FASTQ Preprocessor. Bioinformatics.

[83] Dobin A., Davis C.A., Schlesinger F., Drenkow J., Zaleski C., Jha S., Batut P., Chaisson M., Gingeras T.R. (2013). STAR: Ultrafast Universal RNA-Seq Aligner. Bioinformatics.

[84] Li B., Dewey C.N. (2011). RSEM: Accurate Transcript Quantification from RNA-Seq Data with or without a Reference Genome. BMC Bioinform..

[85] McCarthy D.J., Chen Y., Smyth G.K. (2012). Differential Expression Analysis of Multifactor RNA-Seq Experiments with Respect to Biological Variation. Nucleic Acids Res..

[86] Durinck S., Spellman P.T., Birney E., Huber W. (2009). Mapping Identifiers for the Integration of Genomic Datasets with the R/Bioconductor Package biomaRt. Nat. Protoc..

[87] Tang D., Chen M., Huang X., Zhang G., Zeng L., Zhang G., Wu S., Wang Y. (2023). SRplot: A Free Online Platform for Data Visualization and Graphing. PLoS ONE.

[88] Hart M., Rheinheimer S., Leidinger P., Backes C., Menegatti J., Fehlmann T., Grässer F., Keller A., Meese E. (2016). Identification of miR-34a-Target Interactions by a Combined Network Based and Experimental Approach. Oncotarget.

[89] Rehmsmeier M., Steffen P., Höchsmann M., Giegerich R. (2004). Fast and Effective Prediction of microRNA/Target Duplexes. RNA.

