# Supplementary material for: MALAT1 Expression Is Deregulated in miR-34a Knockout Cell Lines
Source: Noncoding RNA. 2025 Aug 5;11(4):60. doi: 10.3390/ncrna11040060 (PMC12388995; doi:10.3390/ncrna11040060)
Supplement: Supplementary file 1 [file ncrna-11-00060-s001.zip › ncrna-3671700-supplementary.pdf]

Figure S1

A

chr1:9,151,668-9,151,777(-)

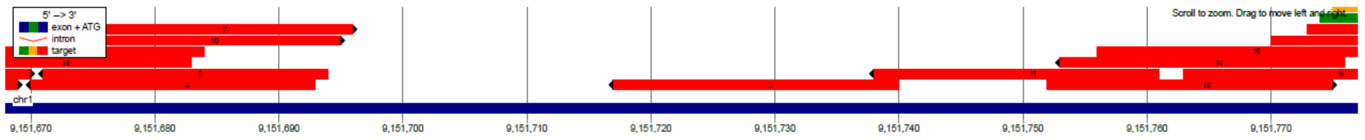

Download results: Please select one

View in UCSC genome browser

Rank	Target sequence	Genomic location	Strand	GC content (%)	Self-complementarity	MM0	MM1	MM2	MM3	Efficiency
1	GCCGGTCCACGGCATCCGGAGGG	chr1:9151775	+	75	3	0	0	0	0	49.86
2	CCGGTCCACGGCATCCGGAGGGG	chr1:9151776	+	75	3	0	0	1	1	57.36
3	GGTTGTTGTGAGCAATAGTAAGG	chr1:9151718	-	40	0	0	0	0	4	46.66
4	TAGAAGTGCTGCACGTTGTGGGG	chr1:9151671	-	50	1	0	0	0	6	57.72
5	CTAGAAGTGCTGCACGTTGTGGG	chr1:9151672	-	50	1	0	0	0	6	43.78
6	GGCCGGTCCACGGCATCCGGAGG	chr1:9151774	+	80	3	0	0	1	6	52.47
7	CACAACGTGCAGCACTTCTAGGG	chr1:9151674	+	50	1	0	0	1	7	42.43
8	ACTCACAGCTGGCCGGTCCACGG	chr1:9151764	+	65	0	0	0	0	9	56.60
9	GCTGGCCGGTCCACGGCATCCGG	chr1:9151771	+	75	2	0	0	2	6	44.57
10	CCACAACGTGCAGCACTTCTAGG	chr1:9151673	+	55	1	0	0	1	8	37.67
11	TTCTTTGGCAGTGTCTTAGCTGG	chr1:9151739	-	45	0	0	0	0	11	42.78
12	TGCACGTTGTGGGGCCCAAGAGG	chr1:9151662	-	65	1	0	0	1	15	59.18
13	CTCGCTTCATCTTCCCTCTTGGG	chr1:9151648	+	50	0	0	0	1	19	26.11
14	GCCAGCTGTGAGTGTTTCTTTGG	chr1:9151754	-	50	0	0	0	2	18	35.57
15	GCCAAAGAAACACTCACAGCTGG	chr1:9151753	+	50	0	0	0	2	23	56.13
16	AAGAAACACTCACAGCTGGCCGG	chr1:9151757	+	50	0	0	0	2	25	64.83
17	TCTCGCTTCATCTTCCCTCTTGG	chr1:9151647	+	50	0	0	0	7	43	33.26

B

```
-     -a  --    -         uU        -   A          -guga  a
ggcc  gc  ugug aguguuucu  GGCAGUGU CUU GCUGGUUGUu      gc a
 ||||  ||  |||| |||||||||  |||||||| ||| ||||||||||       ||
ccgg  ug  gcac ucgugaaga  CCGUCAUA GAA CGACUAACga      ug u
c     gg  uu    g         UC        U   -          aggaa  a
```

C

Pre-miR-34a

NC_000001.11, chr1:9,151,668-9,151,777(-)

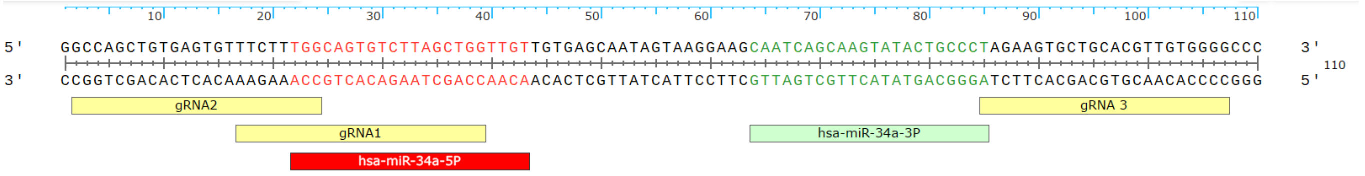

Figure S1 – gRNA design and strategy for miR-34a KO generation

(**A**) CHOPCHOP tool output for the best scoring guide RNAs (gRNAs) targeting the genomic region chr1:9151668-9151777(-) containing the locus for miR-34a. The three guide RNAs selected for the study are highlighted in green. **(B)** Representative structure of the stem-loop precursor pre-miR-34a, according to miRBase (accession MI0000268). Sequences of the mature miR-34a-3p and miR-34a-5p are highlighted in green and red, respectively. **(C)** Full linear sequence of pre-miR-34a (accession MI0000268), showing sites recognized by the three gRNAs selected and the position of the mature miR-34a-3p (green) and miR-34a-5p (red). Image realized using SnapGene Viewer (www.snapgene.com).

Table S1

Table S1 – CHOPCHOP prediction of off-target effects for the three guide RNAs used.

Gene symbol	Gene description	Chromosomal position	Matched sequence (mismatching nt in lower case)	Exonic/ Intronic
**gRNA 1**				
**TMEM132B**	Homo sapiens transmembrane protein 132B	chr12:125188676	TTCTTTGGgAGaGTCTcAGCAGG	Intronic
**RNASEH2B**	Homo sapiens ribonuclease H2 subunit B	chr13:50916823	TTCTTTGGgAGTtcCTTAGCAGG	Intronic
**GNG2**	γ subunit of a guanine nucleotide-binding protein (G proteins)	chr14:51832499	TTCTcTctCAGTGTCTTAGCTGG	Intronic
**SLC14A1**	Homo sapiens solute carrier family 14 member 1 (Kidd blood group)	chr18:45729148	TTCTTTGGtAGTGTCTTAtgTGG	Intronic
**ZNF431**	Homo sapiens zinc finger protein 431	chr19:21150460	TTCTTTGtCAGTtTCTTAaCTGG	Intronic
**gRNA 2**				
**PRRX1**	Homo sapiens paired related homeobox 1	chr1:170738902	CCAAAGAAACAtTCACtGCTGGt	Exonic, not part of CDS (NM_022716.4)
**KCNQ1**	Homo sapiens potassium voltage-gated channel subfamily Q member 1	chr11:2674196	GCCAGtTGTGgGTtTTTCTTGGG	Intronic
**DSCAML1**	Homo sapiens DS cell adhesion molecule like 1	chr11:117680463	GCCAGCTGTGAGaGcTTCTcAGG	Intronic
**BMP4**	Belongs to the TGF-beta family	chr14:53950357	GCCcGCTGTGAGTGaTgCTTAGG	Intronic
**INSR**	Homo sapiens insulin receptor	chr19:7166138	CCCAAGAcACACTCACtGgTGGC	Intronic
**PREX1**	Homo sapiens phosphatidylinositol-3,4,5-trisphosphate dependent Rac exchange factor 1	chr20:48743039	CCGgAGtAACACTCACAGCTGaC	Intronic
**RUNX1**	Alpha subunit of core binding factor (CBF)	chr21:35405509	CCCAAGAAACACaCACAGCTGtg	Intronic
**TMEM40**	Homo sapiens transmembrane protein 40	chr3:12734869	CCCAAGtAcCcCTCACAGCTGGC	Intronic
**UNC13B**	Homo sapiens unc-13 homolog B	chr9:35386318	GCCAGCTGTcAGTGgcTCTTTGG	Intronic
**gRNA 3**				
**PALMD**	Palmdelphin	chr1:99659492	TAGAAGTGCTcCAgGTTcTGGGG	Intronic
**CNTN4**	Homo sapiens contactin 4	chr3:2435853	CCACAgAACGTGgAGCACTaCTA	Intronic

Figure S2

A

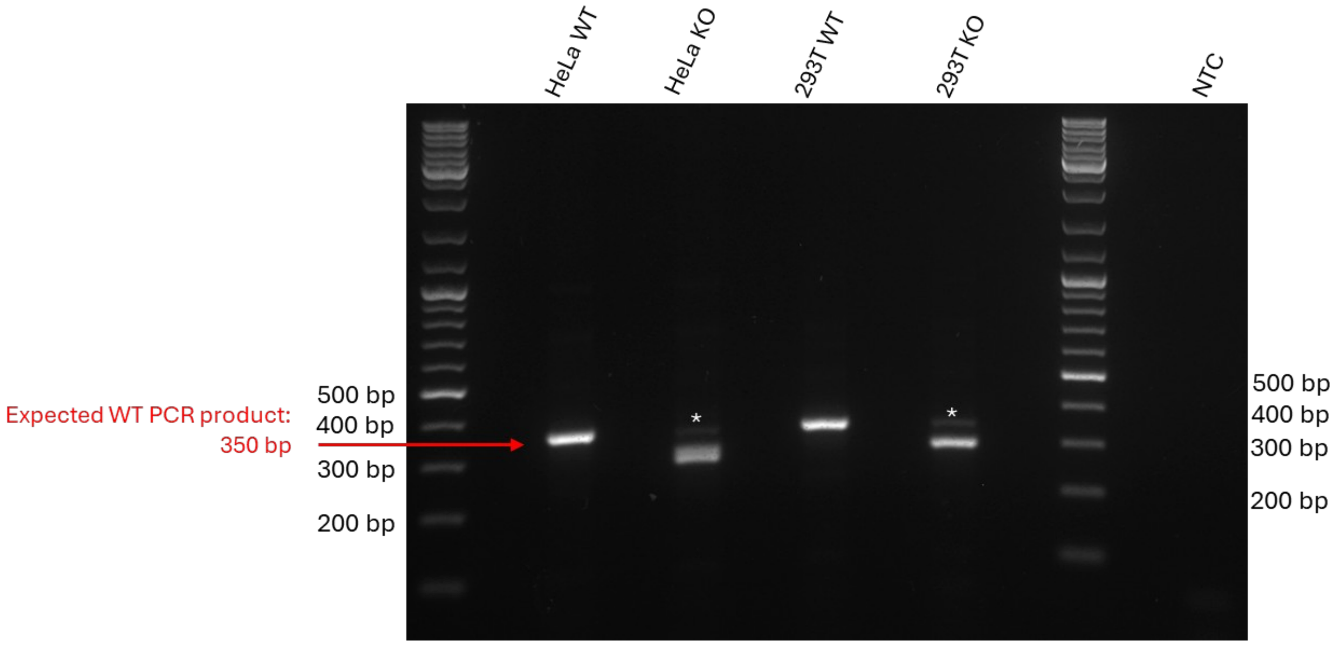

B

```
CLUSTAL O(1.2.4) multiple sequence alignment


Ref-seq            tcggcctcctgcatcctttctttcctccccacatttccttcttatcaacaggtgctgggg      60
miR-34a_WT         tcggcctcctgcatcctttctttcctccccacatttccttcttatcaacaggtgctgggg      60
HeLa_miR-34a_KO    tcggcctcctgcatcctttctttcctccccacatttccttcttatcaacaggtgctgggg      60
293T_miR-34a_KO    tcggcctcctgcatcctttctttcctccccacatttccttcttatcaacaggtgctgggg      60
                   ************************************************************

Ref-seq            agaggcaggacaggcctgtcccccgagtcccctccggatgccgtggaccGGCCAGCTGTG      120
miR-34a_WT         agaggcaggacaggcctgtcccccgagtcccctccggatgccgtggaccGGCCAGCTGTG      120
HeLa_miR-34a_KO    agaggcaggacaggcctgtcccccgagtcccctccggatgccgtggaccGGCCAGCTGTG      120
293T_miR-34a_KO    agaggcaggacaggcctgtcccccgagtcccctccggatgccgtggaccGGCCAGCTGTG      120
                   ************************************************************

Ref-seq            AGTGTTTCTTTGGCAGTGTCTTAGCTGGTTGTTGTGAGCAATAGTAAGGAAGCAATCAGC      180
miR-34a_WT         AGTGTTTCTTTGGCAGTGTCTTAGCTGGTTGTTGTGAGCAATAGTAAGGAAGCAATCAGC      180
HeLa_miR-34a_KO    AGTGTNNNNNNNNNNNNNNNNNNNNNNNNNNNNNNNNNNNNNNNNNNNNNNNNNNNNNNN      180
293T_miR-34a_KO    AGTGTTTCTTTGGCAGTGTCTAAGTGC---------------------------------      147
                   *****

Ref-seq            AGTATACTGCCCTAGAAGTGCTGCACGTTGTGGGGCCCaagagggaagatgaagcgagag      240
miR-34a_WT         AGTATACTGCCCTAGAAGTGCTGCACGTTGTGGGGCCCaagagggaagatgaagcgagag      240
HeLa_miR-34a_KO    NNNNNNNNNNNNNNNAAGTGCTGCACGTTGTGGGGCCCaagagggaagatgaagcgagag      240
293T_miR-34a_KO    ---------------------TGCACGTTGTGGGGCCCaagagggaagatgaagcgagag      186
                                        ***************************************

Ref-seq            atgcccagaccagtgggagacgccaggacttcggaagctct  281
miR-34a_WT         atgcccagaccagtgggagacgccaggacttcggaagctct  281
HeLa_miR-34a_KO    atgcccagaccagtgggagacgccaggacttcggaagctct  281
293T_miR-34a_KO    atgcccagaccagtgggagacgccaggacttcggaagctct  227
                   *****************************************
```

Figure S2 –PCR verification of the presence of deletion in the KO cells and alignment of Sanger sequences from WT and miR-34a KO cells.

(A) Electrophoretic gel of a PCR showing deletions in the MIR34A gene in the KO cell lines, compared to the parent WT lines. Genomic DNA extracted from all cell lines was used as template. The lower bands correspond to DNA with deletions introduced by CRISPR editing. The upper faint band (*) indicates a residual potentially unedited genomic DNA. The presence of two bands in the HeLa KO lane might indicate presence of multiple and different deletions in the cell line. WT = cell lines wild-type for MIR34A locus. KO = cell lines knocked out for MIR34A locus. NTC = no template control. Primers used for the PCR anneal in the MIR34A locus:
Fw: 5'-GATGGAGTCTTGCTAGTTGCCTGG-3'; Rv: 5'-GCAGAAGAGCTTCCGAAGTCCTGG-3'

(B) Alignment of Sanger sequences from WT and miR-34a KO, HeLa and 293T cell lines, with the reference genome sequence mapping in MIR34A locus. The templates for Sanger sequencing consisted in purified PCR products generated from genomic DNA using the primers described in panel (A). The sequence of pre-miR34a is indicated with capital letters (aligned Refseq position 110 to 219). Dashes indicate deletions, while "N" indicates regions with overlapping sequences in the chromosome 1.Ref-seq = NCBI Reference Sequence NC_000001.11:c9151605 - 9151886, reverse strand, Homo sapiens chromosome 1, GRCh38.p14 primary assembly (GCF_000001405.40), that includes the MIR34A locus (NC_000001.11:c9151668-9151777, reverse strand); miR-34a_WT = Sanger sequence of genomic DNA from HeLa miR-34a WT cells; HeLa_miR-34a_KO = Sanger sequence of genomic DNA from miR-34a KO HeLa cells; 293T_miR-34a_KO = Sanger sequence of genomic DNA from miR-34a KO 293T cells. Alignments realised using Clustal Omega (CLUSTAL O – 1.2.4) [81].

Figure S3

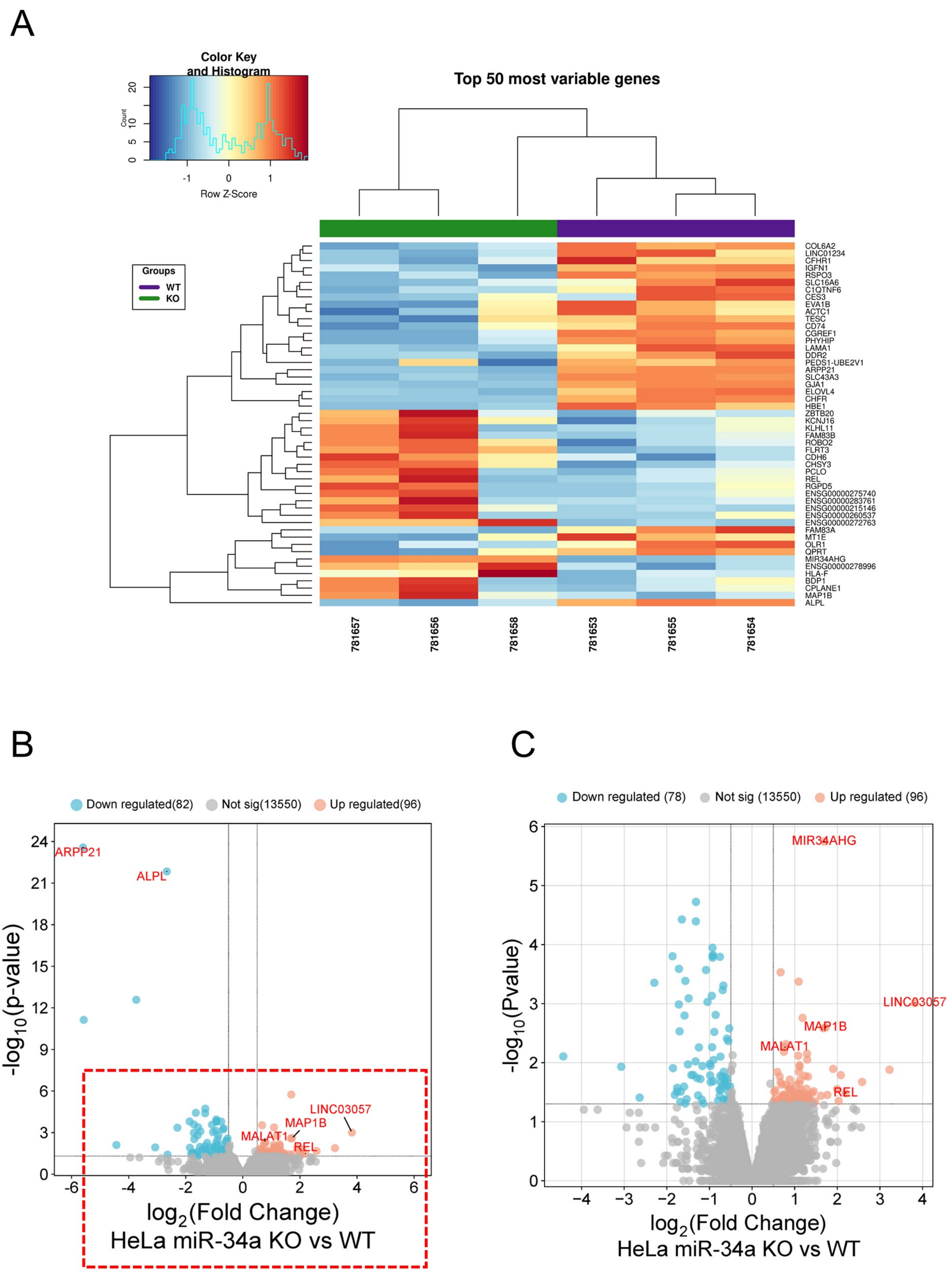
A
Color Key and Histogram
Count
Row Z-Score
Top 50 most variable genes
Groups
WT
KO
COL6A2
LINC01234
CFHR1
IGFN1
RSPO3
SLC16A6
C1QTNF6
CES3
EVA1B
ACTC1
TESC
CD74
CGREF1
PHYHIP
LAMA1
DDR2
PEDS1-UBE2V1
ARPP21
SLC43A3
GJA1
ELOVL4
CHFR
HBE1
ZBTB20
KCNJ16
KLHL11
FAM83B
ROBO2
FLRT3
CDH6
CHSY3
PCLO
REL
RGPD5
ENSG00000275740
ENSG00000283761
ENSG00000215146
ENSG00000260537
ENSG00000272763
FAM83A
MT1E
OLR1
QPRT
MIR34AHG
ENSG00000278996
HLA-F
BDP1
CPLANE1
MAP1B
ALPL
781657
781656
781658
781653
781655
781654
B
Down regulated(82)
Not sig(13550)
Up regulated(96)
-log10(p-value)
ARPP21
ALPL
LINC03057
MAP1B
MALAT1
REL
log2(Fold Change)
HeLa miR-34a KO vs WT
C
Down regulated (78)
Not sig (13550)
Up regulated (96)
-log10(Pvalue)
MIR34AHG
LINC03057
MAP1B
MALAT1
REL
log2(Fold Change)
HeLa miR-34a KO vs WT

Figure S3 – NGS RNA-seq analyses in HeLa WT and miR-34a KO cells.

(**A**) Heatmap comparison of the top 50 most variable upregulated and downregulated genes between HeLa WT and HeLa miR-34a KO. (**B**) Volcano plot showing the differentially expressed genes between HeLa WT and miR-34a KO. The x-axis illustrates the log2 fold changes (FC), and the y-axis indicates the –log10 p-values. The color of the scatter points indicates the changed type of differentially expressed genes (red, up; gray, stable; blue, down). (**C**) Magnification of the volcano plot shown in (**B**), focusing on the red squared area.

Figure S4

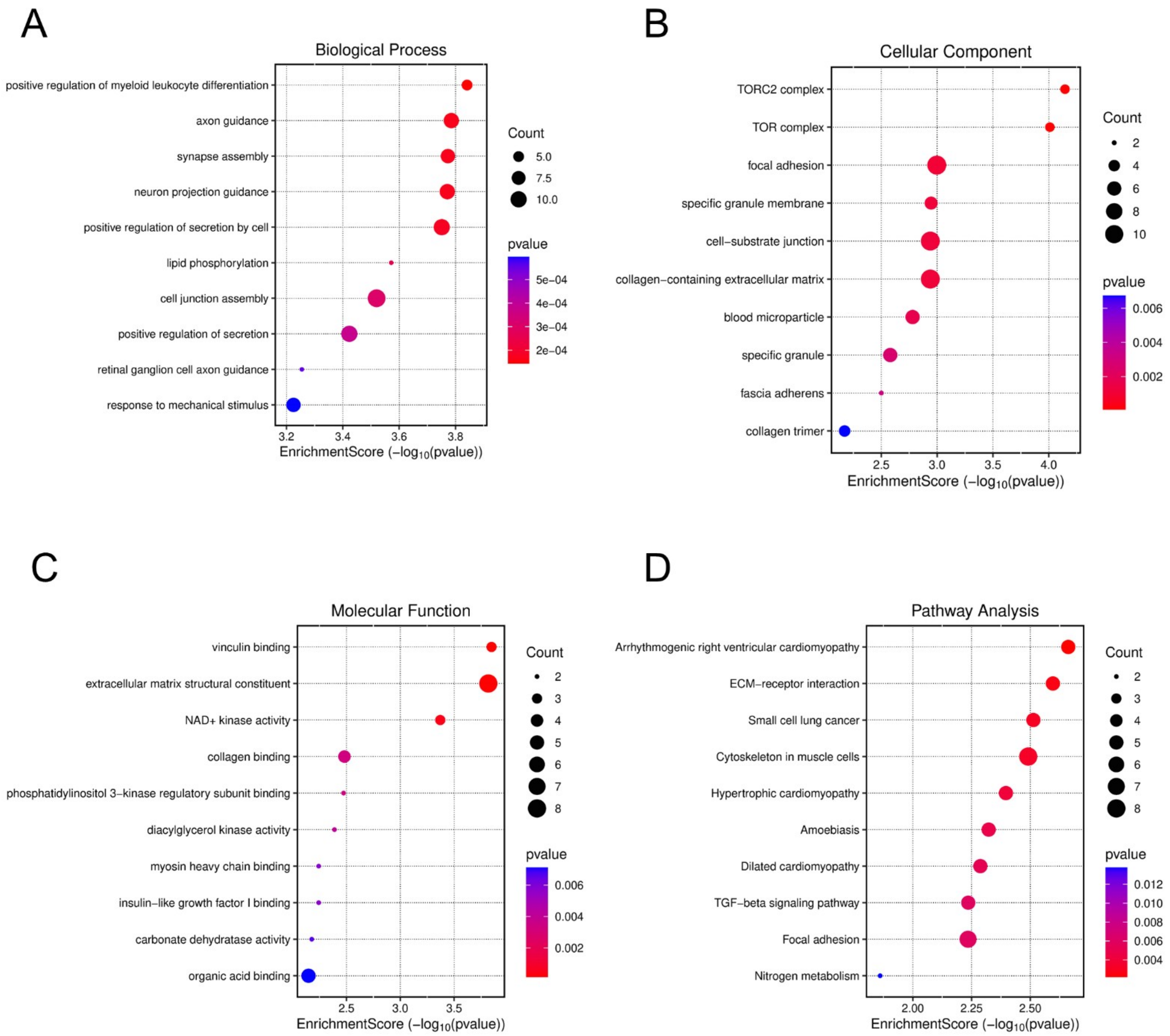

Figure S4. Gene Ontology and KEGG analysis of RNA-seq data.

Bubble plots showing enrichment analyses of GO terms between HeLa WT and miR-34a KO cell lines for biological process (**A**), cellular component (**B**) and molecular function (**C**) and KEGG pathways (**D**).
